# Research trends and prospects on brain metastasis from breast cancer: A bibliometric analysis

**DOI:** 10.3389/fonc.2023.1091249

**Published:** 2023-04-05

**Authors:** Si-qi Wu, Yan Liu, Jie Zhou, Yan-ting You, Xing-hong Zhou, Li-qian Chen, Hiu Yee Kwan, Xiao-shan Zhao, Yi-fen Wu, Yan-yan Liu

**Affiliations:** ^1^ School of Traditional Chinese Medicine, Southern Medical University, Guangzhou, Guangdong, China; ^2^ School of Chinese Medicine, Hong Kong Baptist University, Hong Kong, Hong Kong SAR, China; ^3^ Department of Oncology, Affiliated Dongguan People’s Hospital, Southern Medical University, Dongguan, Guangdong, China

**Keywords:** breast cancer, brain metastasis, bibliometric analysis, research trend, TKI (tyrosine kinase inhibitor), ADC (antibody-drug conjugate)

## Abstract

**Introduction:**

Brain metastasis is the terminal event of breast cancer with poor prognoses. Therefore, this article aimed to provide an updated summary on the development, hotspots, and research trends of brain metastasis from breast cancer based on bibliometric analysis.

**Method:**

Publications on breast cancer with brain metastasis retrieved from the Web of Science Core Collection. CiteSpace, VOSviewer, and other online bibliometric analysis platforms were used to analyze and visualize the result.

**Result:**

In totality, 693 researchers from 3,623 institutions across 74 counties and regions published a total of 2,790 papers in 607 journals. There was a noticeable increase in publications in 2006. The United States was the dominant country with the most publications followed by China. University Texas MD Anderson Cancer Center was the most productive institution, while Dana Farber Cancer Institution was the most cited. *Journal of Neuro-Oncology* published the most papers, while *Journal of Clinical Oncology* ranked first based on cocited analysis. Nancy U. Lin was the most productive and cited author with high influence. There was a focus on basic research, clinical trials, local therapy, treatment optimization, and epidemiological studies regarding brain metastases from breast cancer. References focused on pathogenesis, prevention, treatment, and prognosis were cited most frequently, among which the clinical trial of novel treatment attracted most attention from researchers. Reference citation burst detection suggested that new therapies such as the novel tyrosine kinase inhibitor and antibody–drug conjugate may lead the research trends in the future.

**Conclusion:**

High-income countries contributed more to the field of breast cancer with brain metastasis, while developing countries like China developed quickly. Furthermore, the success of novel therapies in recent years may lead to the new era of treatment of breast cancer with brain metastasis in the future.

## 1 Background

Breast cancer has been the most common malignancy in women. There has been a slight increase in breast cancer incidence rates since 2004 (1), with new cases in women reaching 281,550 in 2021, accounting for 30% of all female cancers (2). Although with a relatively high survival rate that reaches 90%, the mortality of breast cancer still ranked second in female cancer, approximately 90% of which are associated with complications from recurrent or metastatic diseases (3). Unfortunately, even patients with early-stage breast cancer will develop distant metastasis, which accounts for one-third of all cancer cases (4).

The development of breast cancer brain metastasis is regarded as a late event with a worse prognosis compared to metastasis to other organs. Different subtypes of breast cancer vary in the rate of brain metastasis. Triple-negative breast cancer [Triple-negative: hormone receptor (HR)–negative/human epidermal growth factor receptor 2 (HER2)–negative] and HER2+ breast cancer (HR-negative/HER2-positive) have a higher likelihood of developing brain metastasis, with rates of 25%–27% and 11%–20%, respectively. Both luminal A and luminal B subtypes have a lower risk of brain metastasis (l8%–15% and 11%, respectively) (5–7).

Despite the attention of researchers on brain metastasis from breast cancer, there has not yet been a bibliometric analysis reviewing the research output on publications concerning the topic. Herein, we utilized some bibliometric analysis tools to explore the frontiers and hotspots of studies on brain metastasis from breast cancer. Bibliometric analysis employs citation count as an assessment to measure of research quality (8). As a quantitative method, bibliometric analysis helps to trace the research profiles of different countries, institutions, and researchers that promoted the scientific production, behavior, and development in the related fields (9, 10). Bibliometric reviews on breast cancer have covered several topics, focusing on a variety of different treatments including nanomedicine (9), immunotherapy (11), the application of pan-cancer studies in treatment (12), and radiotherapy (13).

In this study, we used CiteSpace and VOSviewer to analyze papers related to breast cancer brain metastasis and summarized the research findings. We examined the evolution and development of research hotspots in the breast cancer brain metastasis from 2006 to 2022, identifying new hotspots and topics. The aim of this study is to contribute new insights and ideas to research of breast cancer brain metastasis in the future.

## 2 Material and methods

### 2.1 Data collection

**Figure 1:**
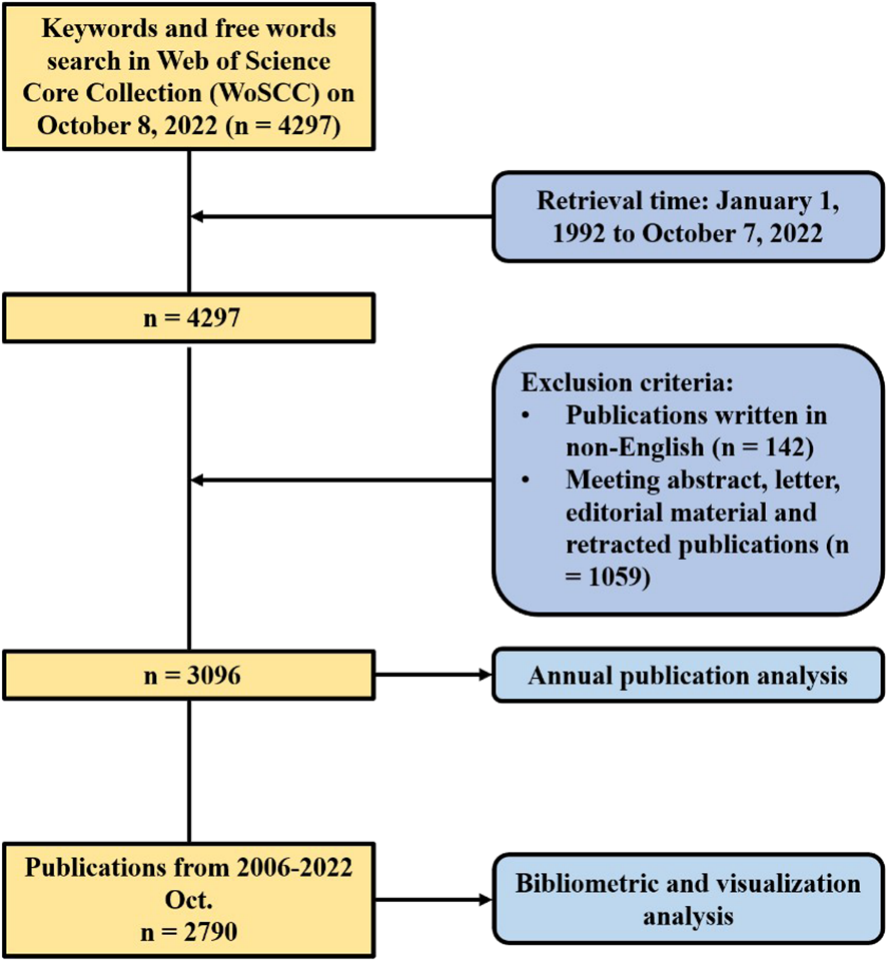
Flowchart of the process of publication selection.

Articles related to brain metastasis from breast cancer were retrieved from the Science Citation Index Expanded (SCIE) of Web of Science Core Collection, which were all published during 1 January 1992 to 7 October 2022. Search strategy was based on the advanced search option with the following strategy: TS = (“breast cancer” OR “breast carcinoma”) AND TS = (“brain metastas*” OR “cerebral metastas*” OR “intracranial metastas*” OR “central nervous system metastas*” OR “secondary brain tumor”). The full records and cited references of the data were extracted and downloaded in a plain text file and a tab-delimited text file, consisting of publication year, authorship, title, abstract, author keywords, citation count, reference, journal title, institution, and country. Only literature written in English was contained in processes of search and downloading, which were completed within 1 day on 8 October 2022 to avoid errors caused by frequent database updates. A total of 3,096 publications were contained in the first set of data (year: 1992–2022). It may be because the data network was so complex that some functions of CiteSpace [version 6.1.R3 (64-bit)] ran very slowly. Therefore, we limited the time from 1 January 2006 to 7 October 2022 with the same strategy of search and selection mentioned earlier. A total of 2,790 publications were contained in the second set of data (year: 2006–2022). Only the annual number of publications was conducted based on the first set of data. Other analyses including country, institution, author, reference, and keyword were based on the second set of data. Occupying 89% of all the 3,096 publications in recent two decades, the analysis of the second set of data cover provided a snapshot of recent research in brain metastasis from breast cancer. **Figure 1** showed the process of publication selection. Furthermore, since this study did not include any animals or experiments, ethical consent was not required.

### 2.2 Data analysis and visualization

Two online platforms and three software were used to analyze and visualize publications related to breast cancer brain metastasis. Datawrapper (https://www.datawrapper.de/) was used to draw the map of the regional distribution of publications and BIBLIOMETRIC (https://bibliometric.com/) was used to analyze the annual publications of the most productive countries as well as the collaboration between different countries. Microsoft Excel 2019 was used to analyze the annual distribution of publications on brain metastasis from breast cancer.

CiteSpace is a visualization and analyzation software designed by Prof. Chaomei Chen. It is used to discover the collaborative network and critical and pivotal points in the scientific literature of a specific topic. The analysis is based on the theory of co-occurrence and cocitation. When two publications are cited together by another publication, there is a cocitation relationship between the two publications. Burst detection is another practical function of CiteSpace to discover emerging words or references by analyzing the change of frequency of their citation or occurrence in a short time. We used CiteSpace to draw a matrix network of authors and cocited authors We also detected references with strong citation burst to find the influential references in the related field. Moreover, we detected keywords with strong occurrence burst and drew a timeline of keyword clusters, which concluded the hotspot development in different year and provided insight on the future trends in the related region. The strength of nodes was calculated of cosine, and pathfinder was used to detect the most representative network.

VOSviewer is another useful software to visualize the network map of scientific papers. We used bibliographic coupling analysis and cocitation analysis to visualize institutions and journals. We also used cooccurrence analysis to draw a cluster network of keywords. To avoid the repeat caused by expression difference, we appended the thesaurus so that the software could recognize terms like “metastasis” and “metastases” as the same term. Additionally, this study did not include any animal or experiments; thus, ethical issues were not required.

## 3 Results

### 3.1 Publication output of research on breast cancer brain metastasis

**Figure 2:**
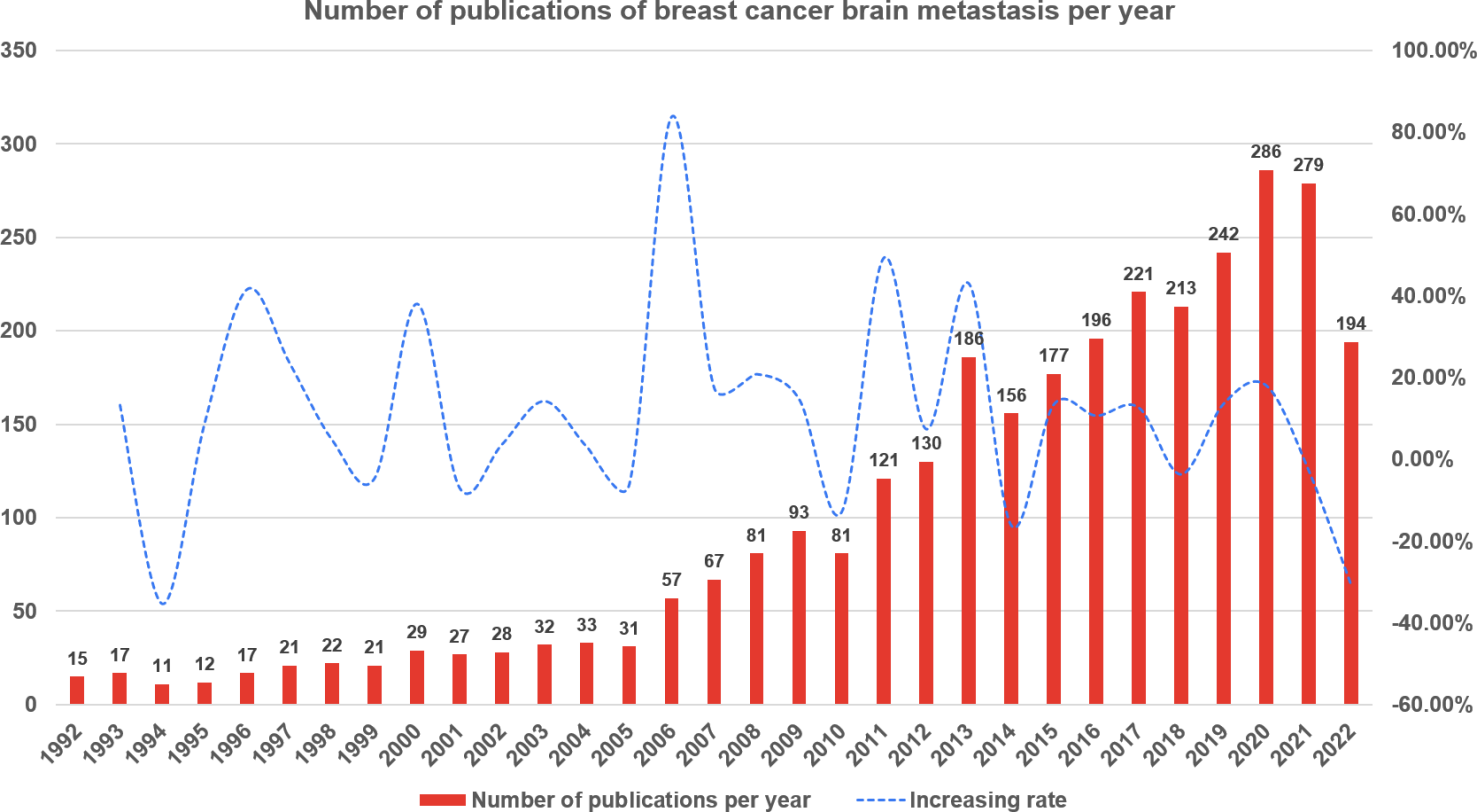
The annual number of publications of breast cancer brain metastasis from 1 January 1992 to 7 October 2022.

Web of Science Core Collection yielded a total of 3,096 publications related to breast cancer brain metastasis in the recent three decades. As demonstrated in **Figure 2**, from 1992 to 2005, the annual output rose slightly with fluctuation. In 2006, the annual number of publications was increased sharply to 57, which almost doubled that of last year. Since then, the number of publications increased substantially in general year by year and peaked in 2020 with a total of 286 publications. For this reason, we used the data from 2006 to 2022 in the follow-up analysis.

### 3.2 Profile of countries and institutions

**Figure 3:**
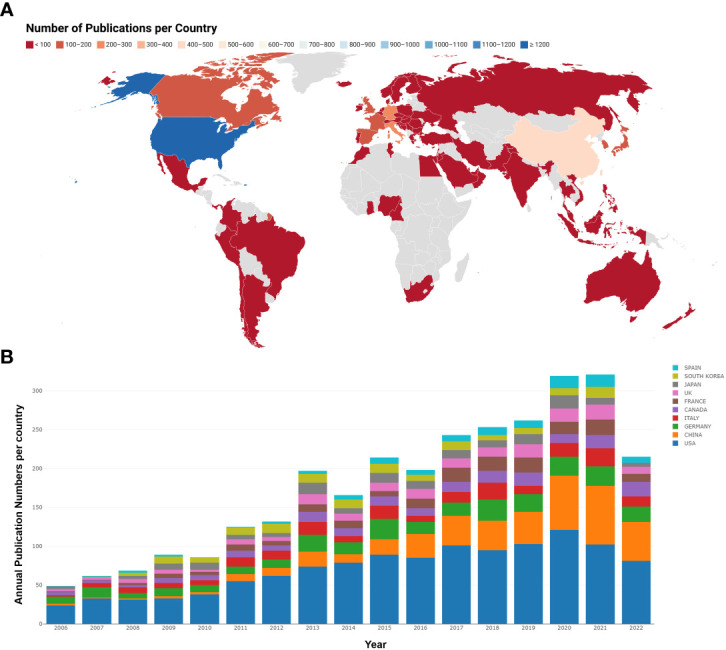
Analysis of country based on the number of publications. **(A)** The number of publications per country. Countries with less publications were colored red, while countries with most publications were colored blue. Countries without publications in the related region were colored gray. **(B)** The annual publication number and trend of the 10 most productive countries. Different countries were represented by different colors, and the height of each color blot reflected the number of publications of a specific country in a specific year.

**Figure 4:**
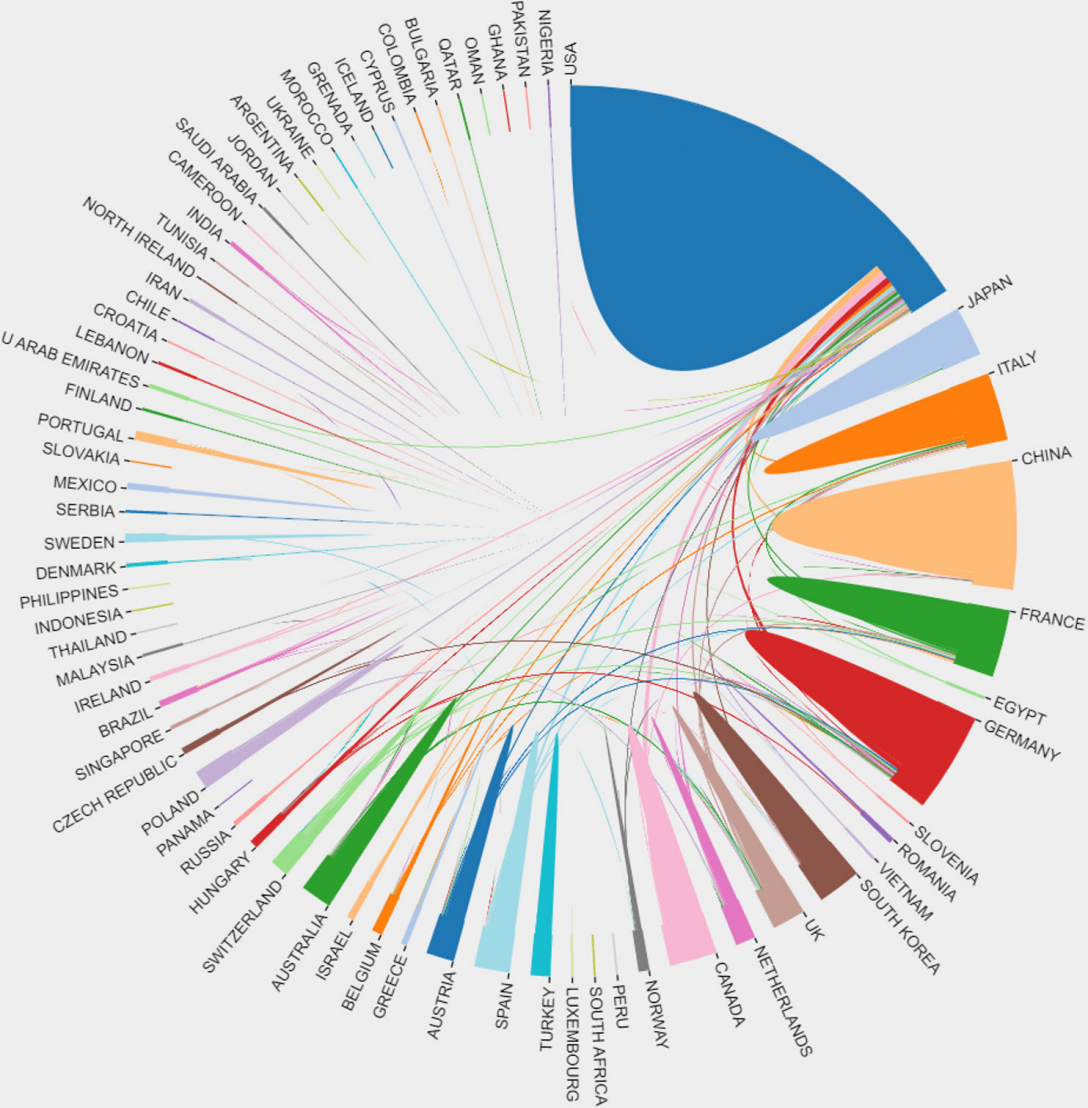
The connection among different countries in brain metastasis from breast cancer. Each country was represented by a fragment on the outer part of the circle. The area of the fragment is proportional to the number of publications of the correspondent country and the size of the arc to the strength of cooperation between two countries.

A total of 74 countries or regions contributed to the studies on breast cancer brain metastasis. As shown in **Figure 3**, the United States was ranked the first and China second and Germany third based on the number of publications. It is worth mentioning that China showed an emerging boost, especially in the recent decade, but the average citation stayed limited (**Table 1**). Regarding research collaboration, the United States had the broadest range of academic collaborations with countries all around the world (**Figure 4**).

**Table 1:** Five most productive countries.

Ranking	Countries	Number of publications	Citation per publication
1	USA	1,215	50.5975
2	CHINA	431	19.0255
3	GERMANY	283	46.6714
4	ITALY	203	40.9458
5	CANADA	176	55.2045

**Figure 5:**
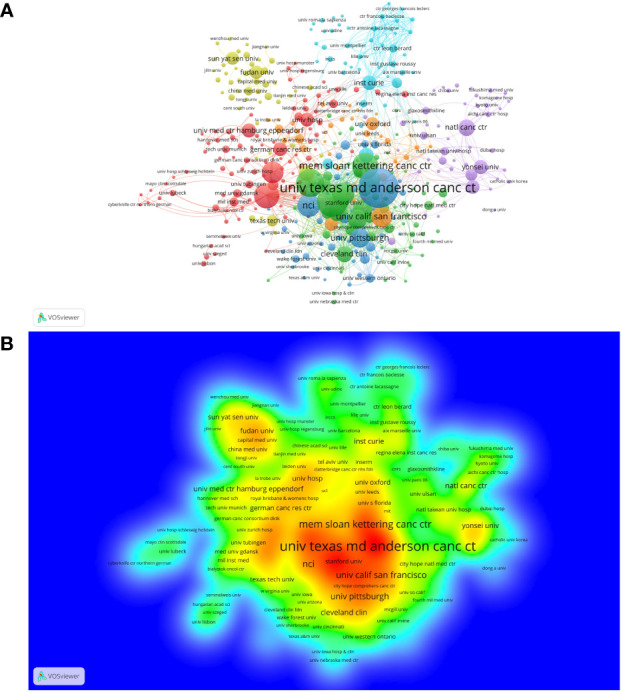
The co-occurrence cluster and production density of institutions. **(A)** cluster network analysis of institutions with at least five publications. The size of the node is proportional to the number of publications of a specific institution. The bigger the node, the more productive the institution. When two institutions occurred in the same publication for three times, there is a link between them. The width of the link represents the strength of co-occurrence between two institutions. Nodes and links in the same color belong to the same cluster, meaning the connections between these institutions are more closer according to the VOSviewer analysis. **(B)** The density map of institutions based on the number of publications. The labels of institutions in red area are the most productive institutions, while labels in the blue area are the less productive ones. From: VOSviewer.

A total of 3,623 institutions participated in the studies of breast cancer brain metastasis, and 404 of them, with at least five publications, are shown in **Figure 5**. All of the 404 institutions were divided into seven clusters based on the co-occurrence analysis. Clusters in deep blue, green, and orange, represented by the University Texas MD Anderson Cancer Center, Dana Farber Cancer Institution and University of California, San Francisco, respectively, were the institutions mainly from America. Institutions in the red cluster were mainly from Europe and America. The yellow cluster mainly consisted of institutions from China, and the purple one consisted of institutions from other East Asia countries. The light-blue cluster mainly consisted of institutions from France. When comparing **Figures 5A, B**, institutions in blue, green, and orange clusters were the most productive ones. Furthermore, as listed in **Table 2**, nine of the most productive institutions were located in America. The University Texas MD Anderson Cancer Center was the most productive institution with a total of 131 publications, while the Dana Farber Cancer Institution was the most influential institution with the highest citation per publication and has the strongest link strength with other institutions.

**Table 2:** 10 most productive and cited institutions.

Institutions	Number of publications	Average citation per publication	Link strength
University Texas MD Anderson Cancer Center	131	61.771	413
Dana Farber Cancer Institution	93	140.2151	478
Harvard University	82	36.9878	256
Memorial Sloan Kettering Cancer Center	77	72.9351	220
Mayo Clinic	72	43.9722	311
National Cancer Institute	64	95.5781	154
Massachusetts General Hospital	62	64.1613	292
Medical University of Vienna	60	39.3667	103
University of California, San Francisco	57	74.7368	407
Northwestern University	52	50.3846	185

### 3.3 Profile of journals, cocited journals

VOSviewer was used to conduct the bibliographic coupling analysis and cocitation analysis of journals. A total of 2,790 papers were published in 607 journals, and 6,628 journals were cited in the references. **Table 3** demonstrated the leading journals in the related research based on publication and citation. *Journal of Neuro-Oncology* published the most papers (n = 111), *Breast Cancer Research and Treatment* second (n = 82), and *Cancers* third (n = 68). Cocitation analysis is an effective method to discover the most influential journals in a specific topic. In the region of brain metastasis from breast cancer, *Journal of Clinical Oncology* ranked first with 9,861 citations, followed by *Clinical Cancer Research* with 4,491 citations and *Cancer Research* with 4257 citations. *Clinical Cancer Research* was the only journal both in top 10 based on production and citation, with the highest impact factor (IF = 13.801) in the list of most productive journals, indicating its special position in the related region.

**Table 3:** 10 most productive and most cited journals.

Journal	Publications	Impact factor	Co-cited journal	Citations	Impact factor
Journal of Neuro-Oncology	111	4.506	Journal of Clinical Oncology	9,861	50.717
Breast Cancer Research and Treatment	82	4.624	Clinical Cancer Research	4,491	13.801
Cancers	68	6.575	Cancer Research	4,257	13.312
Frontiers in Oncology	68	5.738	International Journal of Radiation Oncology*Biology*Physics	4,223	8.013
Oncotarget	57	–	The Annuals of Oncology	3,804	51.769
BMC Cancer	51	4.638	The New England Journal of Medicine	3,623	176.079
Neuro-oncology	47	13.029	Journal of Neuro-Oncology	3,273	4.506
Clinical Cancer Research	46	13.801	The Lancet Oncology	3,001	54.433
PLOS ONE	45	3.752	Cancer	2,725	6.921
International Journal of Molecular Sciences	42	6.208	Nature	2,512	69.504

### 3.4 Profile of authors and cocited authors

**Figure 6:**
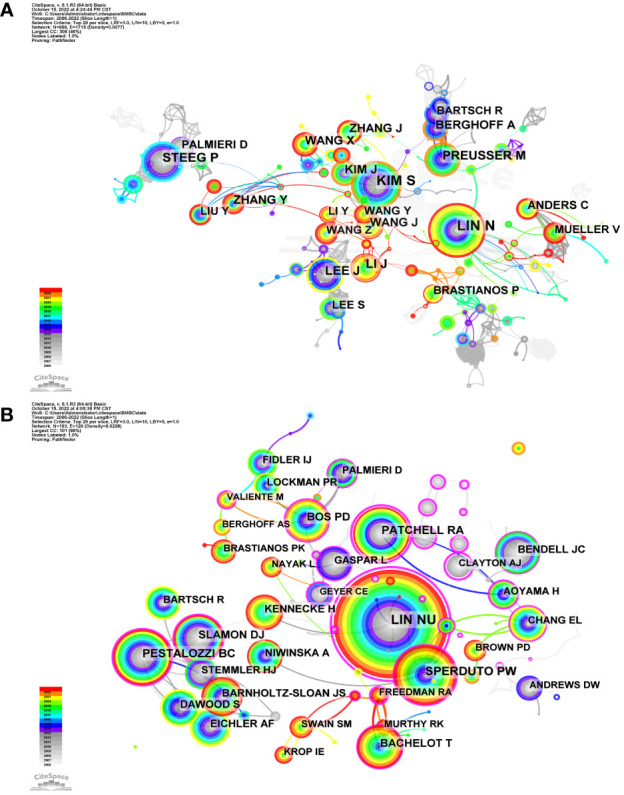
Network of authors and cocited authors on breast cancer brain metastasis. Each tree-ring node represents an author. **(A)** Author network based on cooccurrence analysis. The size of node is proportional to the research output of the author. The color of the tree-ring node represents the publication history of the author. Red tree-ring nodes represent the author who published papers in the recent 2 years, while gray ones represent authors who were productive for a decade. Colorful nodes represent authors with continuous research output. **(B)** Cocited author network based on cocited analysis. The larger the node, the more times the author is cited. Nodes in warmer colors represent authors cited more recently, while nodes in cooler colors represent the opposite.

CiteSpace was also used to visualize the network of authors based on co-occurrence analysis and cocited analysis. In total, 693 authors contributed to the development of research on breast cancer brain metastasis and 994 authors collaborated in the research. The top 20 authors or cocited authors in every year were selected and visualized in the network. The sizes of the nodes are proportional to the number of publications of the author shown in **Figure 6A** and the citation in **Figure 6B**. Links are thicker between two closely collaborated authors. As shown in **Figure 6**, Lin NU, Kim S, Lee J, Berghoff A, and Preusser M were the top five authors based on the number of publications. As shown in **Table 4**, Lin NU was active in this field all over the years with 65 publications in total. In addition, Lin NU was the most cited author based on cocited analysis with 1,000 citations. Sperduto PW ranked second with 468 citations, Pestalozzi BC ranked third with 326 citations, Patchell RA ranked fourth with 315 citations, and Bachelot T ranked fifth with 288 citations. Centrality is used to assess the relationship of one node with other nodes. When the centrality is over 0.1, the node is considered as a landmark node connected closely with other nodes in a network. The centrality of 10 most productive authors all subceeded 0.1. The centrality of 7 authors among the 10 with most citations exceeded 0.1, of which LIN NU (1.24) was the highest. These authors were the leading researchers in the field of brain metastasis from breast cancer and served as a linking bridge with other researchers.

**Table 4:** Top 10 of the most productive authors and cocited authors.

Author	Publications	Centrality	Cocited Author	Citations	Centrality
LIN NU	65	0.04	LIN NU	1,000	1.24
KIM S	54	0.08	SPERDUTO PW	468	0.22
STEEG P	38	0.04	PESTALOZZI BC	326	0.3
PREUSSER M	36	0.04	PATCHELL RA	315	0.99
LEE J	35	0.11	BACHELOT T	288	0.15
LI J	34	0.01	BOS PD	265	0.29
WANG J	30	0.01	SLAMON DJ	255	0.35
ZHANG Y	29	0.1	EICHLER AF	234	0
KIM J	29	0.03	BENDELL JC	231	0.11
BERGHOFF A	29	0	KENNECKE H	231	0

### 3.5 Keywords of breast cancer brain metastasis

**Figure 7:**
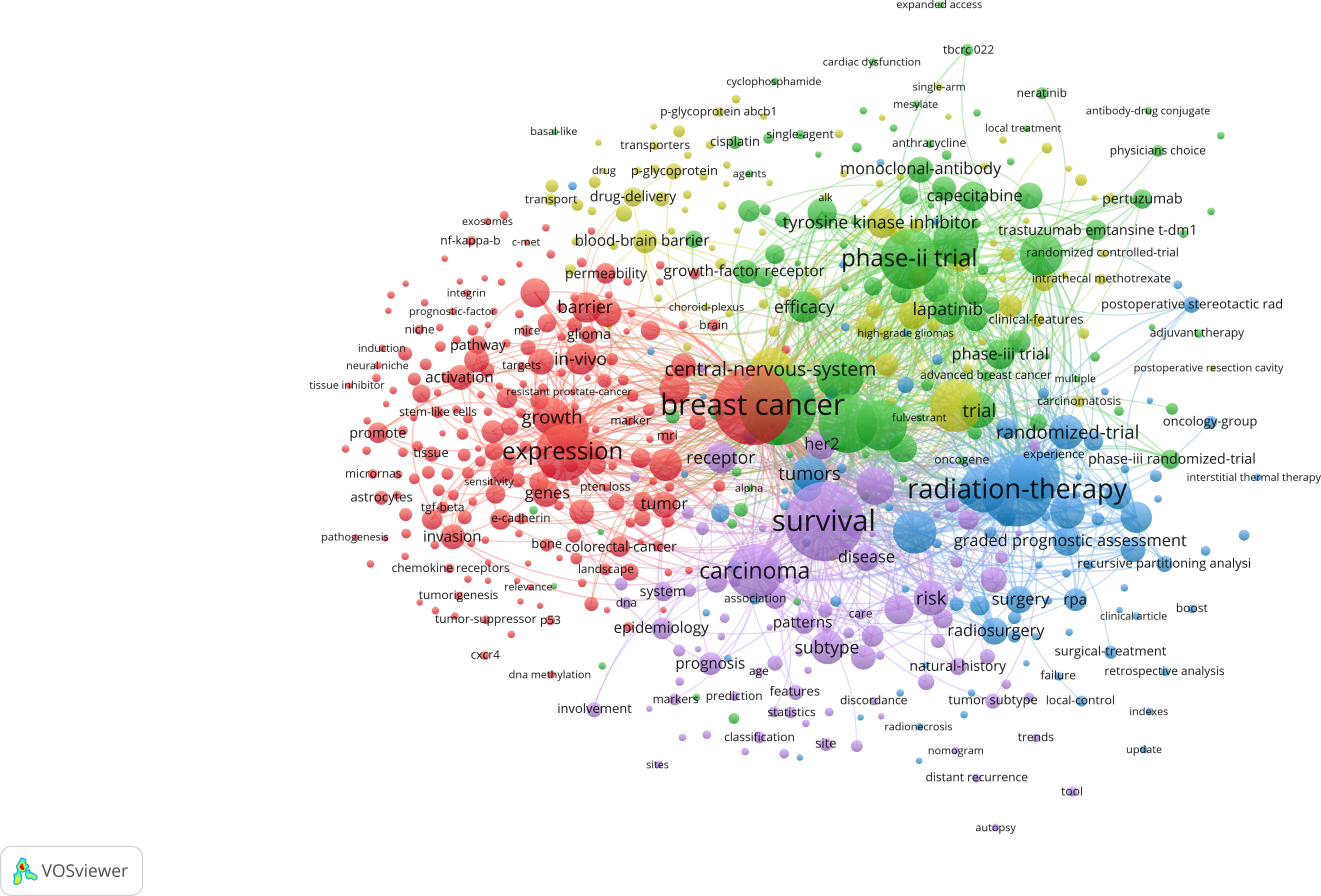
Co-occurrence analysis of keywords during 2006–2022. Lines between two keywords mean the two words coexisted for more than 10 times. The bigger the node, the more frequent it appears. The stronger the line, the more co-occurrences happen. At least 50 keywords are contained in one cluster. Additionally, keywords connected more closely are divided into the same cluster with the same color. From: VOSviewer.

VOSviewer and CiteSpace were used to analyze keywords from different perspectives to provide an overlook of development and trend in the related region. After merging keywords repeated by expression difference, VOSviewer concluded 4,189 keywords and 598 of them occurred more than five times, which were divided into five clusters (**Figure 7**). The red cluster was the largest one with 210 keywords, containing *expression, in-vivo, angiogenesis, microenvironment*, and *endothelial growth-factor*. The green cluster ranked second in size with 201 keywords including *phase II trial, chemotherapy, efficacy, open-label*, and *lapatinib plus capecitabine*. The blue cluster ranked third in size with 97 keywords including *radiation-therapy, stereotactic radiosurgery, prognostic-factors, quality of life, graded prognostic assessment*, and *management*. The yellow cluster contained 96 keywords including *solid tumors, blood-brain barrier, cerebrospinal-fluid, her2-positive breast cancer, drug-delivery*, and *acquired-resistance*. Purple cluster contained 89 keywords including *survival, risk, subtype, diagnosis*, and *recurrence*. Accordingly, keywords were roughly clustered into five categories as follows: basic study, clinical trial of new therapy, local therapy, treatment optimization, and epidemiology.

**Figure 8:**
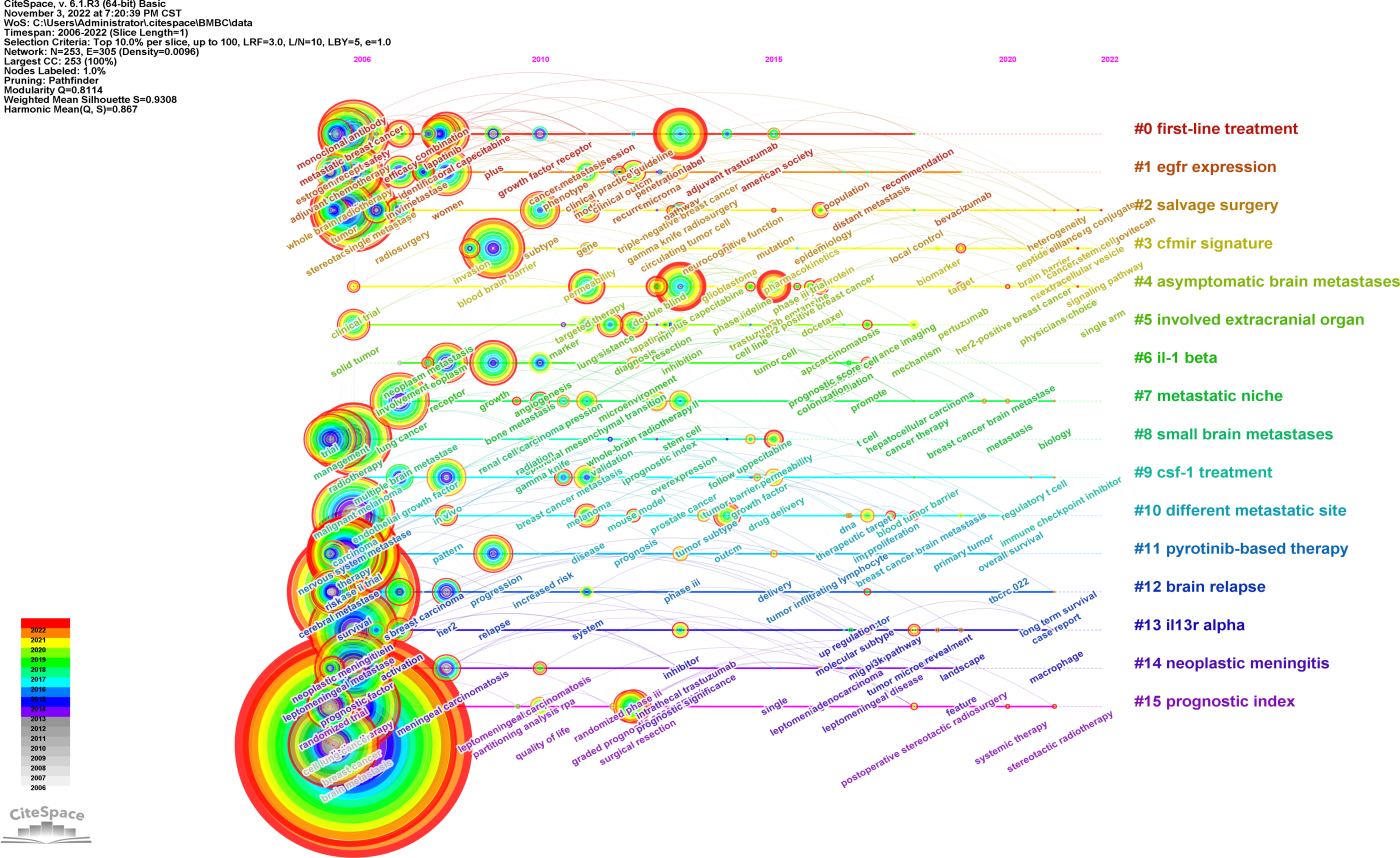
Cluster timeline of keywords of breast cancer brain metastasis. Each tree-ring node represents a keyword. The size of the node is proportional to the time of the occurrence of the keyword. The color of the tree-ring node represents the occurrence history of the keyword. The label of clusters was produced by LLR analysis, and the colors represent different years.

We also used CiteSpace to perform cluster analysis based on the log likelihood ratio (LLR) and drew a timeline of the development of the hotspots in the related region (**Figure 8**). The top 10% of keywords in a year were analyzed, which yielded a network of 253 nodes with 305 links. A cluster with the modularity of 0.8114 and the weighted mean silhouette of 0.9291 was yielded, which meant that the result was highly convincing with a significant cluster structure. There were 15 clusters including first-line treatment, epidermal growth factor receptor (EGFR) expression, pyrotinib-based therapy, the prognostic index, and other clusters, demonstrating the development of research focus on breast cancer brain metastasis.

### 3.6 Cocited references

**Figure 9:**
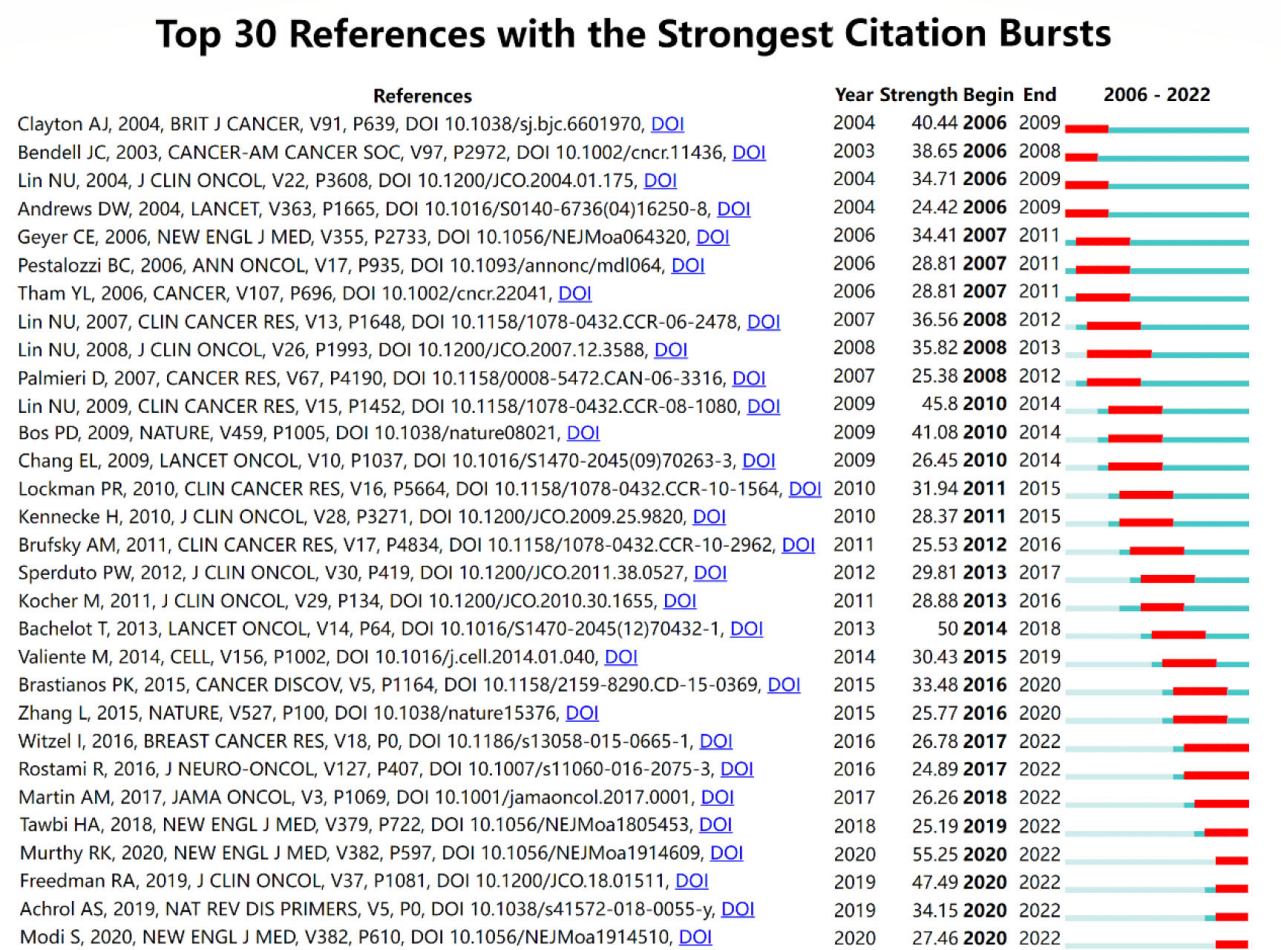
Top 30 references with the strongest citation bursts. The blue line reflects the duration of the citation of the paper. The red segment reflects the duration of the burst.

CiteSpace was used to visualize the network of references of records between 2006 and 2022. The top 5% of the most cited references each year were contained in the cocited analysis, and the network was refined by the pathfinder pruning method. As shown in **Figure 9**, 930 references were detected and 10 of the most cocited publications are listed in **Table 5**. Seven out of the top 10 co-cited publications were clinical trials, namely, five randomized controlled trials, one population-based cohort, and one retrospective cohort. The other most cocited publications included one genome wide association study (GWAS) study, one basic study, and one review. The most cited publication was “Lapatinib plus capecitabine in patients with previously untreated brain metastases from HER2-positive metastatic breast cancer (LANDSCAPE): a single-group phase 2 study (14)” by Bachelot T et al., which was cited 160 times. It is worth mentioning that the sources of these publications strongly overlapped with the list of the most cited journals, which confirmed the importance of these references and journals in the studies of breast cancer brain metastasis.

**Table 5:** Top 10 cocited references.

Year	Author	Title	Journal	Citation
2013	Bachelot T et al. (14)	Lapatinib plus capecitabine in patients with previously untreated brain metastases from HER2-positive metastatic breast cancer (LANDSCAPE): a single-group phase 2 study	The Lancet Oncology	160
2020	Murthy RK et al. (15)	Tucatinib, Trastuzumab, and Capecitabine for HER2-Positive Metastatic Breast Cancer	The New England Journal of Medicine	124
2009	Lin NU et al. (16)	Multicenter Phase II Study of Lapatinib in Patients with Brain Metastases from HER2-Positive Breast Cancer	Clinical Cancer Research	115
2019	Freedman RA et al. (17)	TBCRC 022: A Phase II Trial of Neratinib and Capecitabine for Patients with Human Epidermal Growth Factor Receptor 2–Positive Breast Cancer and Brain Metastases	Journal of Clinical Oncology	113
2015	Brastianos PK et al. (18)	Genomic Characterization of Brain Metastases Reveals Branched Evolution and Potential Therapeutic Targets	Cancer Discovery	105
2016	Witzel I et al. (19)	Breast cancer brain metastases: biology and new clinical perspectives	Breast Cancer Research	102
2009	Bos PD et al. (20)	Genes that mediate breast cancer metastasis to the brain	Nature	96
2012	Sperduto PW et al. (21)	Summary Report on the Graded Prognostic Assessment: An Accurate and Facile Diagnosis-Specific Tool to Estimate Survival for Patients with Brain Metastases	Journal of Clinical Oncology	93
2015	Krop IE et al. (22)	Trastuzumab emtansine (T-DM1) versus lapatinib plus capecitabine in patients with HER2-positive metastatic breast cancer and central nervous system metastases: a retrospective, exploratory analysis in EMILIA	The Annuals of Oncology	91
2017	Martin AM et al.	Brain Metastases in Newly Diagnosed Breast Cancer: A Population-Based Study	JAMA Oncology	90

Burst detection is an effective method to find out the hotspots in different times and conclude the developmental route of a specific field supported by CiteSpace. The publication of the phase II trial of tucatinib, trastuzumab, and capecitabine for HER2-positive metastatic breast cancer (15) gained the most attention from researchers in the related field (burst strength: 55.25). The publications of another two clinical trials focused on the effects of neratinib plus capecitabine (17) and lapatinib (16) separately was ranked the second (burst strength: 47.49) and third (burst strength: 45.8), respectively. The recent publication that most appealed researchers’ attention was “Trastuzumab Deruxtecan in Previously Treated HER2-Positive Breast Cancer (23)”, whose citation can be dated from 2020 with an increasing trend.

## 4 Discussion

### 4.1 General trends

With reference from the number of publications, the publication trend of the breast cancer brain metastasis research field can be divided into two stages. From 1992 to 2005, the publications increased slowly with fluctuation. It may be due to the following two reasons. The first one is that radiotherapy was the mainstream of treatment for brain metastasis and the controversial effect of chemotherapy (24) limited the development of clinical trials. The second one is that patients with brain metastasis were always excluded from clinical trials in this period and medical oncologists were reluctant to leave the advanced patients with brain metastasis without other therapy but just the target therapy of clinical trial, which make it difficult to have enough number of patients and assess the efficacy of chemotherapy (25). In 2005, several randomized trials have confirmed the efficacy of chemotherapy in treating brain metastasis (24, 26–28) and more clinical trials were ongoing, especially those focused on trastuzumab (28), which may lead to the publication boosted in 2006. From 2006 onward, the number of publications in breast cancer brain metastasis increased gradually.

In this study, we summarized the research collaboration in different dimensions as well as research focus and hotspots to understand the development and trend of breast cancer brain metastasis. A total of 693 researchers from 3,623 institutions in 74 counties and regions attributed 2,790 publications in 607 journals, indicating that breast cancer brain metastasis attracted wide attention from researchers all over the world. The United States was the leading country in the related field based on the analysis of institutions and authors’ publications and citations. Moreover, the institutions in the United States exhibited a high degree of collaboration with research institutions from all around the world, especially those from Europe. On the other hand, China had emerged as the most promising country in the last decade. Although the academic influence and research collaboration are still limited, researchers and institutions in China demonstrate diligent effort to promote the development of studies on breast cancer brain metastasis. Furthermore, regionally limited collaborations were observed based on institution analysis. Spatial isolation caused by the COVID-19 pandemic has increased the development of online meetings, which serve as a means to break the spatial boundaries between countries and accelerate the research development significantly.

Journal and cocited journal analysis listed the most productive and influential journals in the related field. The 10 top productive journals covered both basic study and clinical study, while half of the top 10 cited journals focused on clinical research, which corresponded to the result of the cocited analysis of references. It implies that clinical research has an important clinical significance in breast cancer brain metastasis. In our study, we list the 10 most productive authors and 10 most frequently cited authors. These authors contributed to the foundation of the related region. The most productive and cited author was Nancy U. Lin, who contributed 65 publications in the field. She has led and participated in numerous clinical trials on breast cancer brain metastases (16, 29, 30) and has participated in the development of guidelines in this field (31, 32). In addition, Nancy U. Lin was the one with highest centrality in top 10 cocited authors, which showed her great influence in the related field.

Cocitation analysis is used to evaluate the relevance between papers, and papers with higher cocitation were considered as the milestones of a specific region. Analyzing the publications cocited most frequently helped to set up the basic knowledge and research focus of breast cancer brain metastasis. The cocited paper ranked first, second, third, fourth, and ninth based on citations were all phase 2 trials of different therapies for patients with brain metastasis from HER-2 positive breast cancer. As mentioned before, HER2-positive is the breast cancer subtype that most frequently develops brain metastasis when compared to other subtypes. Former studies found that the active therapy of HER-2 positive breast cancer, trastuzumab, seemed to increase the risk of brain metastasis. Therefore, new therapy was innovated to solve these clinical problems, including lapatinib plus capecitabine, tucatinib plus trastuzumab and capecitabine, lapatinib, neratinib plus capecitabine, and trastuzumab emtansine (T-DM1). “Lapatinib plus capecitabine in patients with previously untreated brain metastases from HER2-positive metastatic breast cancer (LANDSCAPE): a single-group phase 2 study” published by Bachelot T et al. in *The Lancet Oncology* was the most cited paper (160 citations). This study has confirmed the efficacy of lapatinib plus capecitabine as the first-line treatment of HER-2-positive breast cancer brain metastasis (14). The second cited paper was “Tucatinib, Trastuzumab, and Capecitabine for HER2-Positive Metastatic Breast Cancer” published by Murthy RK et al. in *The New England Journal of Medicine* in 2020. The novel HER-2 inhibitor, tucatinib, demonstrated inspiringly active efficacy in patients with brain metastasis from HER2-positive breast cancer. Progression-free survival in the first year was 24.9% in the tucatinib-combination group and 0% in the placebo-combination group (15). The fourth paper was “TBCRC 022: A Phase II Trial of Neratinib and Capecitabine for Patients with Human Epidermal Growth Factor Receptor 2–Positive Breast Cancer and Brain Metastases” published by Freedman RA et al., which confirmed the efficacy of neratinib plus capecitabine against refractory HER2-positive breast cancer brain metastasis (17). The ninth paper confirmed the capacity of T-DM1 to lengthen the overall survival of patients with HER2-positvie breast cancer brain metastasis when compared with lapatinib plus capecitabine treatment. The fifth cited paper was “Genomic Characterization of Brain Metastases Reveals Branched Evolution and Potential Therapeutic Targets” published by Brastianos PK et al., which clarified the value of sequence of primary biopsies by detecting alterations in the assistance of the prediction of distant metastasis including brain metastasis (18). The eighth cited paper was an evaluation of different graded prognostic assessment (GPA) indices *via* multi-institutional retrospective analysis (21). The seventh cited paper was the only basic study in the 10 most cited papers, which anchored COX2, HBEGF, and ST6GALNAC5 as the key genes mediating breast cancer brain metastasis (20). These references can be divided into categories: pathogenesis, prevention, treatment, and prognosis of breast cancer brain metastasis, among which treatment was the most dominant area.

### 4.2 Focus and hotspots

Keyword analysis was employed to identify the trends of development and research hotspots in the related fields. The co-occurrence analysis of VOSviewer divided the keywords into five clusters. We divided the five clusters into the following five categories: basic study, clinical trial, local therapy, treatment optimization, and epidemiology. Keywords with high frequency highlighted the focus within the branch category. In the red cluster, *expression, cell, in vivo*, and *growth* were the most common keywords, which were the fundamental elements of basic studies. The green cluster contained keywords such as *phase II trial, trastuzumab, efficacy, open-label*, and *lapatinib plus capecitabine*. The LANDSCAPE trial set the lapatinib plus capecitabine as the first-line therapy, and more clinical trials were registered and conducted. The combination of target therapy and chemotherapy was the mainstream in clinical trials, which complemented each other clinically (33–36). Local therapy was represented by the blue cluster, which included keywords such as *radiation-therapy, stereotactic radiosurgery, surgical resection, prognostic-factors, management*, and *graded prognostic assessment*. Local therapies remained the cornerstone of treatment for patients with brain metastasis from breast cancer. These therapies include surgical resection, stereotactic radiotherapy, and whole brain radiotherapy. The approach chosen for treatment is individualized based on the extent and characteristics of the brain metastasis (37, 38). For patients with endocrine-resistant breast cancer who develop brain metastasis and are resistant to most chemotherapies, surgical tumor resection and stereotactic radiotherapy have demonstrated efficacy in improving overall survival and reducing symptoms associated with brain metastasis, which remains crucial in optimizing outcomes for this population (39). Keywords in the yellow cluster were mainly related to treatment optimization including *nanoparticles* and *focused ultrasound*, which were used in preclinical trials, and the multidisciplinary crosstalk seemed to improve the efficacy of treatment at present (40, 41). The purple cluster was mainly related to epidemiology with keywords such as *survival, risk, subtype, diagnosis*, and *recurrence*. The development of research trends reflected by keywords were sorted out from the cluster timeline.

Burst detection was an effective method to discover the hotspots and identify potential future developmental trends. In this study, we found the references with citation burst. The burst of four references began in 2020, and the burst is still ongoing, including three clinical trials and a review. The first clinical trial was the phase II trial of tucatinib, trastuzumab, and capecitabine for HER2-positive metastatic breast cancer (15), earning the most attention of researchers in the related field (burst strength: 55.25) within 2 years. As mentioned earlier, tucatinib demonstrated apparent positive efficacy, especially in patients with brain metastasis, which was a milestone of the treatment of breast cancer brain metastasis with a tyrosine kinase inhibitor (TKI). Another clinical trial burst in 2020 was “TBCRC 022: A Phase II Trial of Neratinib and Capecitabine for Patients With Human Epidermal Growth Factor Receptor 2–Positive Breast Cancer and Brain Metastases,” which provided new possibility to the treatment of refractory HER2-positive breast cancer brain metastases (17). The third clinical trial burst recently was “Trastuzumab Deruxtecan in Previously Treated HER2-Positive Breast Cancer,” A novel antibody–drug conjugate (ADC), trastuzumab deruxtecan, showed durable antitumor activity in patients with HER2-positive metastatic breast cancer (23). The outstanding success of treating HER2-positive breast cancer and other solid tumors may represent a new era of tumor treatment (42). The last reference burst in 2 years was a review that concluded the molecular mechanisms and clinical therapies of brain metastasis (43), which provided an overview of the current understanding of brain metastasis from preclinical and clinical perspectives.

In conclusion, our analysis of keywords and citation bursts provides insight into trends and hotspots in research on brain metastasis from breast cancer. The success of novel therapies, such as TKIs and ADCs, may represent a new era of treatment in patients suffering brain metastasis from breast cancer. Our findings suggest that future research in this region may focus on these novel therapies and optimizing treatment approaches for brain metastasis.

## 5 Strengths and limitations

We summarized the developmental route of the research on breast cancer brain metastasis from January 2006 to October 2022. However, there are also some limitations in our bibliometric study. First, the analysis was based on publications retrieved from the SCIE of Web of Science Core Collection, which did not have papers indexed by other databases such as Scopus and Google. Second, a reference to a document can be either confirmatory or contradictory, which would result in bias in the number of citations. Third, searching based on title, abstracts, and keywords means that some of the manuscripts that involved breast cancer brain metastasis might not be included.

## 6 Conclusion

In conclusion, there has been a general increase in the annual number of publications on breast cancer brain metastasis from 2006 to 2022. The main findings are as follows:

- Institutions from all over the world participated in the study of breast cancer brain metastasis. The United States was ranked first in both the number of publications and institutions. The University Texas MD Anderson Cancer Center was the most productive institution, while the Dana Farber Cancer Institution was the most cited. However, the research collaboration between countries and institutions was regionally limited.
- The *Journal of Neuro-Oncology* published the most papers (n = 111), while the *Journal of Clinical Oncology* ranked first with 9,861 citations based on cocited analysis.
- Nancy U. Lin was the most productive and cited author with high influence in the field.
- Research on breast cancer brain metastasis was focused on the basic study, clinical trial, local therapy, treatment optimization, and epidemiology.
- The most cited references were focused on pathogenesis, prevention, treatment, and prognosis, among which treatment attracted the most attention. New therapies developed rapidly in the recent 3 years, and the treatment of brain breast cancer metastasis got a breakthrough with novel TKI and ADC-based therapies, which may be the mainstream treatment in the future.

## Data availability statement

The original contributions presented in the study are included in the article/supplementary material. Further inquiries can be directed to the corresponding authors.

## Author contributions

S-QW and YL were responsible for the conception and design of the research. Y-TY, X-HZ, L-QC and HK contributed to the data collection and filter. S-QW and JZ participated in writing the manuscript. X-SZ revised this manuscript critically for intellectual content. This research is administrative supported by Y-FW and Y-YL. All authors contributed to the article and approved the submitted version.
